# Natural selection on sleep duration in *Drosophila melanogaster*

**DOI:** 10.1038/s41598-020-77680-0

**Published:** 2020-11-26

**Authors:** Caetano Souto-Maior, Yazmin L. Serrano Negron, Susan T. Harbison

**Affiliations:** Laboratory of Systems Genetics, Systems Biology Center, National Heart Lung and Blood Institute, Bethesda, MD USA

**Keywords:** Evolution, Genetics

## Abstract

Sleep is ubiquitous across animal species, but why it persists is not well understood. Here we observe natural selection act on *Drosophila* sleep by relaxing bi-directional artificial selection for extreme sleep duration for 62 generations. When artificial selection was suspended, sleep increased in populations previously selected for short sleep. Likewise, sleep decreased in populations previously selected for long sleep when artificial selection was relaxed. We measured the corresponding changes in the allele frequencies of genomic variants responding to artificial selection. The allele frequencies of these variants reversed course in response to relaxed selection, and for short sleepers, the changes exceeded allele frequency changes that would be expected under random genetic drift. These observations suggest that the variants are causal polymorphisms for sleep duration responding to natural selection pressure. These polymorphisms may therefore pinpoint the most important regions of the genome maintaining variation in sleep duration.

## Introduction

Sleep is a highly conserved behavior that has been observed in mammals, birds, fish, reptiles, amphibians, and invertebrates^1^. While highly conserved across species, sleep can vary radically among individuals within a species. The amount of time spent sleeping, for example, varies with genotype^2–13^. Genetically identical individuals can also vary in their sleep times, and the degree of discordance also has a genetic component^3,14^. Further, fluctuations in sleep duration often occur from day-to-day in a single individual^15–18^ and are partly genetic^15,18^. The reasons for this tremendous variability in such a broadly conserved behavior remain obscure, though its partial genetic basis suggests that variation in sleep is maintained by some combination of past natural selection, mutation, and random genetic drift^19^.

Previous work supports the idea that variation in sleep is maintained by natural selection, though the studies are scarce. Nor is the form(s) of natural selection that might maintain variation in sleep-stabilizing selection, balancing selection, or directional selection-known. Recent evidence suggests that sleep varies clinally in *Drosophila melanogaster*^20,21^, indicating that it may be a target of natural selection. Average nightly sleep bout length in flies, and to a lesser extent, average daily sleep bout length is negatively correlated with latitude^20,22^. Clinal differences in sleep duration persist among fly strains that have been maintained in a laboratory environment for years^21^, as would be expected if sleep is shaped by natural selection. Furthermore, clinal distributions of polymorphisms have been observed in genes associated with sleep and circadian behavior^23–27^. In addition, many of the genes affecting sleep have conserved functional roles in sleep across species^28–30^. This work promotes the idea that sleep is related to organismal fitness, and is therefore a target of natural selection.

Another way to detect natural selection on a complex trait is to breed extreme trait values using artificial selection. Often the response to artificial selection for high or low values of a trait will be greater for one direction than for the other. This asymmetrical response has been observed in many complex traits among *Drosophila* species, including food consumption, the ability to recover from cold stress, cuticular hydrocarbon profiles, wing shape, sex comb number, bristle number, phototaxis, geotaxis, sleep, and diurnal preference^31–39^. One explanation for the asymmetry is that the trait is linked in some way to fitness^40^. Asymmetry in the response to artificial selection can have other causes, however, including but not limited to random drift, inbreeding depression, maternal effects, and genes with large effects^19,40^. Additional evidence for natural selection can be detected by relaxing the constraints of an artificially selected trait so that natural selection, if present, is free to modify the trait^19^. Relaxed artificial selection constraints revealed fitness relationships governing both light and heavy 8-week body weight in chickens, phototaxis, geotaxis, and desiccation in *Drosophila*^37,41,42^, pleiotropic fitness effects on wing shape^43^, and natural selection effects antagonistic to both sexual selection and development^33,34,44^. Here we relaxed selection on four artificially selected long and short sleeping populations, which enabled us to detect potential effects of natural selection on sleep. Sixty-two generations of relaxed selection resulted in more moderate sleep. Allele frequency changes in key loci exceeded the changes expected under random genetic drift in short-sleeping populations. These comparisons enabled us to distinguish the signal of natural selection and the key genomic variants involved in the phenotypic changes.

Recently, the genetic basis of sleep was hypothesized to be unlike that of circadian rhythm behaviors, which map to a core set of genes regulating centralized feedback loops in defined clock neurons^45^. Large numbers of genes appear to regulate sleep, and their mechanism of action is not confined to a single small region of the brain^45^. Despite the complexity this implies, genes involved in the evolution of sleep include those variants responding to natural selection that we identify here, and have a key role as they are closely related to organismal fitness.

## Results

### Relaxed selection altered sleep duration

Previously, we used artificial selection to generate flies with extreme long and short night sleep duration^38^. Using flies from an outbred population, we selected two replicate populations for short sleep, two for long sleep, and two replicate populations were maintained as an unselected control. In the course of that experiment, we maintained the artificial selection for 13 generations^38^. This breeding scheme generated long and short sleepers that slept an average (± SD) 681.4 ± 45.1 min per night and 77.2 ± 91.9 min per night, respectively, with unselected control populations averaging 425.9 ± 169.4 min^38^.

**Figure 1:**
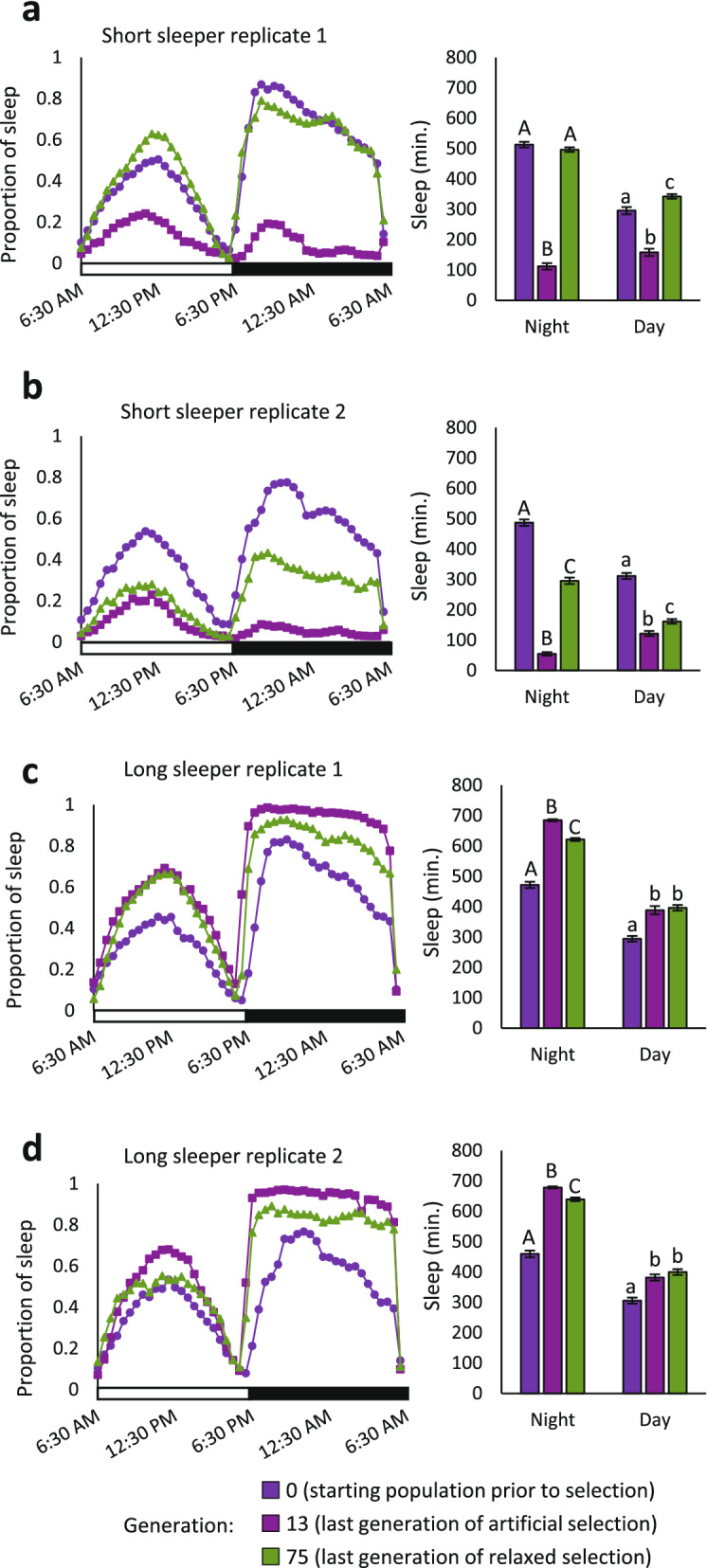
Sleep measured at Generation 75 shows the response to 62 generations of relaxed selection. Sleep duration in the last generation of relaxed selection, Generation 75, is contrasted with that in Generation 0, the starting unselected population, and that of Generation 13, the last generation of artificial selection. The mean proportion of night and day sleep duration are plotted in 30-min bins across time for all flies in the experiment. The white bar on the x-axis indicates day, while the black bar indicates night. Mean night and day sleep duration are quantified in the bar charts. Error bars are standard errors of the mean. (**A**) short sleep replicate 1 (n = 165, 102, and 198 flies in generations 0, 13, and 75, respectively). (**B**) short sleep replicate 2 (n = 199, 158, and 194 flies in generations 0, 13, and 75, respectively). (**C**) long sleep replicate 1 (n = 159, 153, and 193 flies in generations 0, 13, and 75, respectively). (**D**) long sleep replicate 2 (n = 199, 190, and 191 flies in generations 0, 13, and 75, respectively). Means with different letters are significantly different by post-hoc Tukey test (*P* < 0.05).

We then relaxed selection by allowing the flies within each replicate population to mate randomly. We noted that after 47 generations, there was an increase in sleep in the short sleepers^38^. We extended this observation with an additional 15 generations of relaxed selection while maintaining the same parental size of 25 pairs. After 62 generations of relaxed selection, night sleep continued to increase in the short sleepers. Night sleep increased by an average 384.4 min in the replicate 1 population, from 111.9 ± 108.5 min to 496.3 ± 110.0 min (Fig. 1A), and at generation 75 was virtually indistinguishable from that observed at the beginning of the artificial selection experiment. Likewise, there was an average increase of 240.6 min in the replicate 2 short sleeper population when selection was relaxed, from 54.8 ± 71.1 min to 295.4 ± 150.0 min (Fig. 1B). In the long sleeper populations, sleep decreased slightly by 63.6 and 39.1 min, from 685.0 ± 41.4 min to 621.5 ± 68.0 min in replicate 1 and from 678.5 ± 47.7 min to 639.4 ± 83.6 min in replicate 2 (Fig. 1C,D). While differences in male and female night sleep are often present in flies^3,46–49^, we did not observe sex-specific differences in the response to relaxed selection, which was consistent with the response to artificial selection^38^. Relaxing selection for night sleep also evoked a correlated response in day sleep for short-sleeping flies. Day sleep increased by 184.4 and 39.8 min in replicate 1 and replicate 2 short sleeper populations, respectively (Fig. 1A,B), while day sleep at generation 75 was not appreciably different from that at generation 13 in the long sleeper populations (Fig. 1C,D). Thus, natural selection opposed both extreme long and short sleep duration, with the greatest effects observed in the replicate 1 short sleepers.

**Figure 2:**
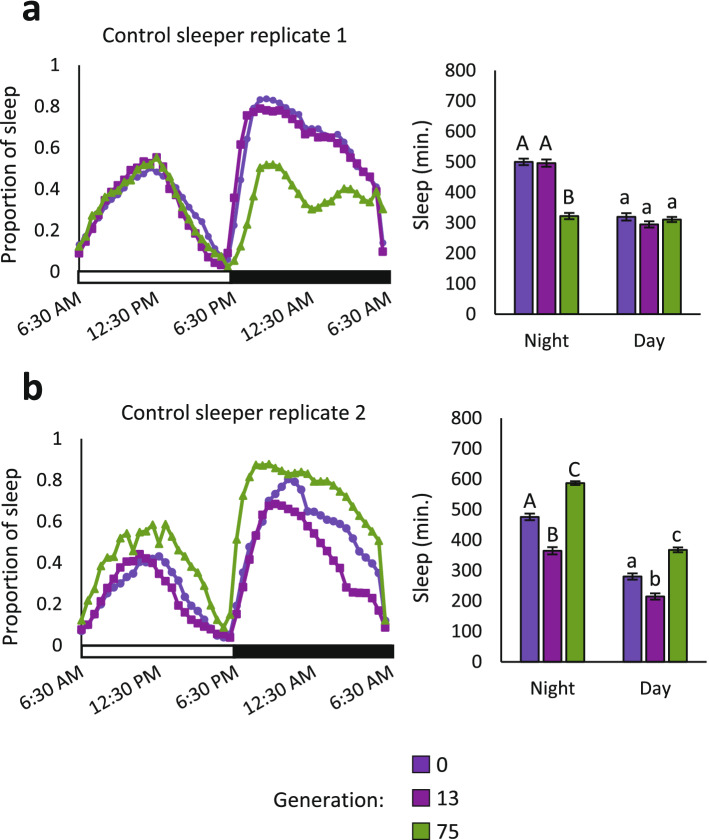
Sleep changes over time in unselected control populations. Sleep duration in the unselected controls is contrasted at Generation 75, Generation 13, and Generation 0. The mean proportion of night and day sleep duration are plotted in 30-min bins across time for all flies in the experiment; mean night and day sleep are quantified in bar charts. Error bars are standard errors of the mean. (**A**) control replicate 1 (n = 164, 161, and 184 flies in generations 0, 13, and 75, respectively). (**B**) control replicate 2 (n = 197, 185, and 189 flies in generations 0, 13, and 75, respectively. Means with different letters are significantly different by post-hoc Tukey test (*P* < 0.05).

Sleep duration changed significantly in the unselected control populations over time as well. Unlike the long and short sleepers, however, sleep drifted in opposite directions for each replicate population. Night sleep decreased on average by 173.4 min in replicate 1, yet increased by 222.2 min in replicate 2, (Fig. 2A,B). Day sleep did not change for replicate 1 but increased by 153.3 min in replicate 2 (Fig. 2B). As no artificial selection was imposed on the control populations, the changes in sleep duration are presumably due to random genetic drift.

Other sleep characteristics changed when selection was relaxed. Sleep latency, the number of minutes it takes to fall asleep at the beginning of the night cycle, increased after artificial selection for short sleep^38^. Under relaxed selection, sleep latency dropped dramatically (Supplementary Fig. S1, Supplementary Table S1). Similarly, artificial selection for long night sleep produced long average night bout lengths which subsequently decreased when selection was relaxed. Other sleep traits showed little response or were less consistent across replicate populations. Thus, traits that changed dramatically with artificial selection for night sleep also reversed course with relaxed selection, suggesting a shared genetic architecture.

### Allele frequencies of target polymorphisms reversed course when selection was relaxed

**Figure 3:**
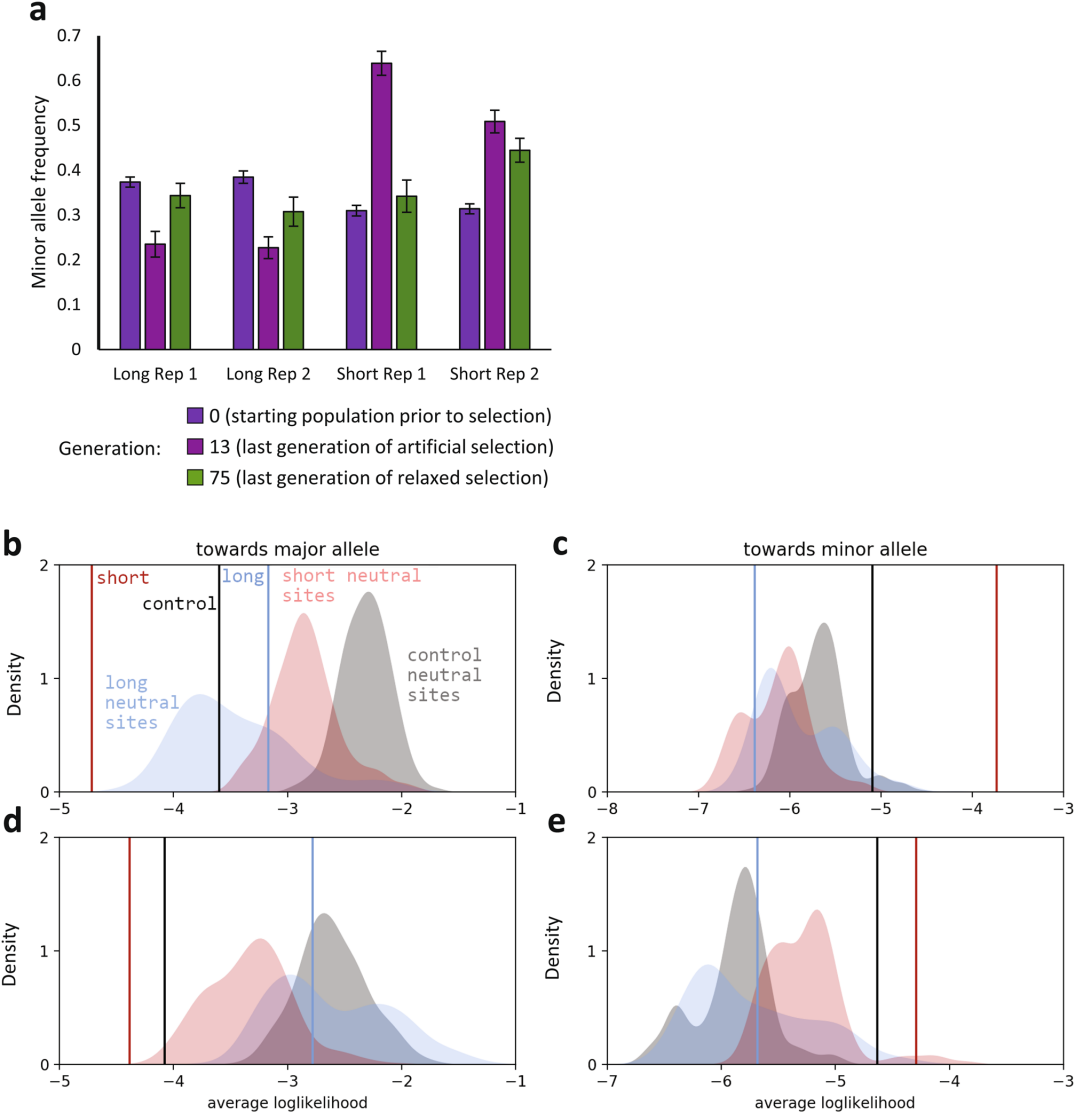
Allele frequency changes in sleep polymorphisms suggest that natural selection acts to maintain moderate sleep duration. (**A**) Generation 0 shows average minor allele frequencies in the starting unselected population; Generation 13 shows average minor allele frequencies in the last generation of artificial selection; and Generation 75 shows average minor allele frequencies in the last generation of relaxed selection. All allele frequencies are calculated relative to the minor allele as defined in Generation 0 of the SAIP. (**B**–**E**) The average loglikelihood for each population for random drift is plotted as a vertical line in relation to the distribution of loglikelihoods of random drift obtained by sampling 126 random (i.e., putatively neutral) polymorphisms 1000 times from the Generation 12 and Generation 75 sequences. Allele frequencies are split according to those that changed towards the major allele from Generation 12 to Generation 75 and those that changed towards the minor allele from Generations 12 to Generation 75. (**B**,**C**) replicate 1 populations; (**D**,**E**) replicate 2 populations. Red indicates the short sleeper mean likelihood and corresponding distribution of neutral polymorphisms; blue indicates the long sleeper mean likelihood and corresponding distribution of neutral polymorphisms; and black indicates the control mean likelihood and corresponding distribution of neutral polymorphisms.

Given the reverse in sleep duration to relaxed selection, we hypothesized that the frequency of alleles that changed in response to artificial selection for sleep duration would also reverse course when selection was relaxed^50^. We sequenced the DNA from these populations at generation 75, after 62 generations of relaxed selection, and assessed the allele frequencies of 126 polymorphisms previously identified as responding to selection for increased and decreased sleep duration^38^. The frequencies of the alleles at these polymorphic loci diverged between long and short sleepers over time with increasing generations of artificial selection^38^. Prior to artificial selection, the minor allele was defined as the least frequent allele at generation 0 in the starting population, the Sleep Advanced Intercross Population (SAIP; see Methods for additional detail), while the major allele was the most frequent allele^38^. During the artificial selection process, the frequencies of the minor alleles tended to increase in lines selected for short sleep, while the frequencies of the major alleles tended to increase in lines selected for long sleep, suggesting that particular haplotypes were associated with long or short sleep^38^. Once selection was relaxed, the allele frequencies in both long and short sleeper populations reversed direction. Minor allele frequencies tended to decrease in short sleepers, while major allele frequencies decreased in long sleepers (Fig. 3A). The changes were pronounced in the replicate 1 short sleeper populations. Thus, the allele frequencies of these polymorphisms became more moderate with relaxed selection, just as the extreme long and short sleep duration became more moderate.

These directional changes in allele frequency could potentially be due to natural selection, random genetic drift, or the combined action of both forces. To distinguish among these possibilities, we first asked whether the changes we observed were significantly different from generation 12, the last sequenced generation of artificial selection. While some polymorphisms appeared to have reached fixation by generation 12 and could not change in allele frequency, particularly in the long sleeper populations (where 14.3% and 24.6% of the alleles were fixed in replicate 1 and replicate 2, respectively), most of the 126 polymorphisms had significant changes in allele frequency in each population (Supplementary Table S2). These changes in allele frequency suggest a dynamic response to relaxed selection. However, significant allele frequency changes also occurred in the control populations, demonstrating a non-negligible contribution of random drift. Allele frequency changes due to the action of natural selection were therefore confounded with changes due to random drift.

To distinguish natural selection from random drift, we compared the changes in allele frequencies that we observed to what would be expected under conditions of random genetic drift alone. We used the Wright–Fisher model of genetic drift to calculate the likelihood of each allele frequency change we observed as given by the theoretical distribution. Under this model, higher likelihoods indicate frequencies compatible with random drift, while lower likelihoods suggest the action of natural selection (i.e., the observed allele frequency change has a low probability of occurrence according to the distribution obtained under random drift). We compared the average likelihood for each population with the likelihood distribution of 1000 sets of 126 randomly chosen (i.e., putatively neutral) polymorphisms from each replicate population (Fig. 3B–E). For polymorphisms where the minor allele frequency decreased (i.e., migrated towards the major allele), the average likelihood of short sleepers of replicate 1 is very low compared to the distributions based on random sampling (Fig. 3B), suggesting that natural selection is more likely than pure drift for these polymorphisms. Short sleepers of replicate 2 fell just outside the random distribution as well (Fig. 3D). In contrast to short sleepers, the average likelihood is much higher for long sleepers, suggesting that these minor allele frequency changes are indistinguishable from drift. For increasing minor allele frequencies (i.e., migration towards the minor allele), the average likelihoods for all populations cannot be distinguished from that expected under a pure drift model (Fig. 3C,E). Unselected controls show more extreme likelihoods than expected from the distribution of randomly-selected sites, although allele frequencies in the outbred starting population (the SAIP), which was maintained for 102 generations, did not deviate from random drift (Supplementary Fig. S2). Thus, natural selection altered sleep duration in short sleepers, particularly replicate 1 short sleepers.

One additional possibility is that de novo mutations occurred in the populations during relaxed selection that might alter sleep. We used LoFreq to search for rare polymorphic variants^51^. Since these populations originated from the *Drosophila* Genetic Reference Panel (DGRP), we eliminated all known DGRP polymorphisms from the data^52^. For the remaining polymorphisms, we searched for those that had arisen in one replicate population only during relaxed selection (see Methods for additional detail). We found 1071 potential de novo mutations: 48 SNP (51 indels) and 254 SNPs (70 indels) in replicate 1 and replicate 2 short sleepers, 98 SNPs (14 indels) and 162 SNPs (23 indels) in replicate 1 and replicate 2 long sleepers, and 133 SNPs (31 indels) and 162 SNPs (25 indels) in replicate 1 and replicate 2 controls, respectively (Supplementary Table S3). These genomic changes could potentially affect sleep.

## Discussion

Relaxing selection resulted in more moderate sleep duration in populations previously selected for long and short sleep, but particularly for the short sleeping populations. This demonstrates the action of natural selection on sleep, and links sleep to fitness. The robust response of short sleep to relaxed selection is consistent with previous observations of instability in short sleep compared to long sleep^3^, and the greater phenotypic response to artificial selection for short sleep^38^. The presumptive reduction of fitness in short sleepers may explain why flies with short-sleeping alleles accrue genetic background modifiers over time that mask the short-sleeping phenotype^53,54^. Alternatively, the reduced response of long sleep may be due to greater numbers of fixed sleep-relevant alleles. Previously, we observed no differences among long and short sleepers for two fitness-related traits, lifespan and egg-to-adult viability^38^. But that leaves many other fitness-related traits potentially connected with sleep. Sleep deprivation in young male flies results in defects in adult courtship behavior, for example^55,56^. Changes in sleep also increased with artificial selection for starvation resistance^57^. Random genetic drift also impacts sleep, which obscures any detectable signal of natural selection. Indeed, much of the phenotypic change we observed was indistinguishable from drift, particularly for the long sleeping populations. Furthermore, mutagenesis and mutation accumulation studies suggest that sleep has a large mutational target^58–60^; we noted 1071 putative de novo mutations accumulating in these populations. These mutations, coupled with random drift, set up a scenario in which large phenotypic changes in sleep are possible in the absence of natural selection, though selection may subsequently act on emerging mutations. Despite this additional complexity, we observed polymorphisms with allele frequency changes that reversed course in response to relaxed selection, with additional support given by the low probability of obtaining these patterns by drift alone, strongly implicating them as causal variants.

We do not yet know what form of selection acts on sleep. Stabilizing selection favors intermediate phenotypes but reduces genetic variation by shifting allele frequencies towards fixation^19,61^. Accordingly, the phenotypic variance is also expected to decrease with each generation of stabilizing selection^62^. A previous mutation accumulation study found evidence for stabilizing selection for sleep^59^. However, most of the sleep-relevant polymorphisms have not reached fixation, and phenotypic variance increased in both short and long sleepers during relaxed selection. Thus, while sleep duration shifted towards more intermediate values after relaxed selection, we did not observe a convincing signature of stabilizing selection. Overall, these data suggest a combination of selective forces, a profile previously observed in complex morphological and behavioral traits^63–66^.

The likelihoods we calculated assumed that allele frequency changes at a given polymorphism occur independently. This is unlikely to be the case. Linkage disequilibrium (LD) could be present among polymorphisms proximal to one another on a given chromosome. LD would be likely in cases where the polymorphisms were 200 bp or less apart^67^. Only three of the polymorphisms we identified were less than 200 bp apart. However, we previously observed that the average distance among selected polymorphisms in high LD (r^2^ > 0.8) remained relatively constant instead of decaying with increased generations of selection as expected^38^. Thus, LD blocks may have been maintained with relaxed selection.

It is plausible that having additional replicate populations might have increased our ability to detect the signal of natural selection^68^; furthermore, the restricted numbers of parents in each generation of relaxation may have enhanced the effects of drift. Alternatively, a large number of haplotypes possible among the 126 sites may be associated with an advantageous sleep phenotype. Thus, a complex gene regulatory system such as this with several dozen components may be capable of achieving a similar output phenotype for many haplotype combinations. This is characteristic of polygenic traits, where disparate genotypes can produce equivalent phenotypes. More precise consideration of these hypotheses requires formulating mechanistic models of the interaction of these mostly unknown components, which is very much beyond the scope of this work. Validation of specific functions of genes will in turn allow mechanistic modeling of the gene-regulatory networks involved and make way for the construction of an accurate genotype–phenotype map.

The seven polymorphisms with the lowest loglikelihoods in the short sleeper replicate 1 population (i.e., the lowest 5%) include four intergenic polymorphisms. Two of the intergenic polymorphisms, 20,754,192 and 20,755,775, are within the same intergenic region of chromosome *2R*. The closest gene, *CG34202*, is a gene with unknown function 453 bp away. The other two intergenic polymorphisms are on chromosome 2L: 4,117,417 and 8,762,348; no genes are within 1000 bp of either of these polymorphisms. The remaining three polymorphisms lie within *ringmaker* (intron), *Fkbp14* (intron), and *CG34176* (3′-UTR). *ringmaker* has a role in neural development: it regulates microtubule formation necessary for proper organization and growth of the synapses at the larval neuromuscular junction^69^. *Fkbp14* reduces *Notch* signaling^70^, which in turn can affect the response to sleep loss^71^. *CG34176* is a computationally predicted gene of unknown function. Additional studies are needed to delineate the function of each polymorphism in sleep.

In conclusion, relaxing artificial selection in four extreme-sleeping populations enabled us to detect the action of natural selection against extreme sleep duration, particularly against extremely short sleep duration. Random genetic drift and newly arising mutations may also impact sleep duration as the mutational target size for sleep is large. However, it is possible to distinguish the underlying genomic signal of natural selection from drift and mutation. This signal of natural selection identifies the most important genomic variants maintaining variation in sleep duration.

## Materials and methods

### Relaxing selection in artificially selected populations

We relaxed selection in the four populations created in Harbison et al.^38^ by maintaining four populations and two control populations in standard environmental conditions. The six populations used in this study were originally created from the Sleep Advanced Intercross Population (SAIP)^38^. The SAIP was constructed from the *Drosophila* Genetic Reference Panel by crossing 5 lines with the shortest night sleep and 5 lines with the longest night sleep in a full diallel and allowing the resulting population to randomly mate for 21 generations with a census size of ~ 800 flies. Additional detail on the construction of the SAIP can be found in^38^.

Each generation we measured sleep in 100 virgin males and 100 virgin females in each population. To select for short sleep, we used the 25 shortest sleeping males and the 25 shortest sleeping females as parents for the next generation. Similarly, we selected for long sleep by choosing the 25 longest-sleeping males and females to be parents for the next generation. We maintained unselected control flies by randomly choosing 25 males and 25 females as parents for the next generation.

Each generation, 25 males and 25 females from each population were allowed to mate at random for 3–5 days. Adults were then cleared from the bottles and fertilized eggs were reared to the adult stage. This process was repeated for 62 generations. Flies were maintained in an incubator at 25 °C, 60% humidity, and 12 h:12 h light:dark cycle. Flies were reared in standard Bloomington Stock Center food (https://bdsc.indiana.edu/information/recipes/bloomfood.html).

### Sleep measurements

Virgin males and virgin females were collected from each population bottle at generation 75. Prior to measuring sleep, flies were aged in same-sex vials with 20 flies per vial to control for mating and social exposure effects on sleep^48,72^. Sleep was measured in 100 males and 100 females per population. We loaded each fly into a *Drosophila* Activity Monitor (DAM2) tube (Trikinetics, Waltham, MA). We recorded sleep and activity from the monitors for 6 days, discarding the first day of data as flies are acclimating to the tubes and recovering from CO_2_ anesthesia . We visually inspected each fly to ensure it survived the experiment. We discarded the data from flies that did not survive all 6 days. Sleep parameters such as sleep duration, number of sleep bouts, and average sleep bout length were calculated for both day and night using Sleep Analysis v6.1 software (R. Sean Barnes). Sleep Analysis v6.1 was also used to calculate sleep latency, the number of minutes before a fly’s first sleep bout at night and waking activity, the number of activity counts per minute spent awake.

### Analysis of sleep phenotypes

Sleep phenotypic differences among generations 0, 13, and 75 were evaluated for each replicate selection population separately using the following fixed ANOVA model:

$$Y = \mu + Gen + Sex + Gen \times Sex + \varepsilon ,$$

where *Gen* is generation. Post-hoc Tukey analysis was used to distinguish differences in sleep among any two generations.

### DNA collection and sequencing

Two samples of 30 female flies per replicate selection population at generation 75 were collected and flash-frozen. We extracted their DNA using the same protocol specified in^38^. We homogenized the flies in 300 µl of cell lysis solution along with four to six 2.38 mm metal beads in an Omni Bead Ruptor (Omni International; Kennesaw, GA) for 5 s of 5 cycles ON/ 5 s OFF at 5 m/s (3100 RPM). An additional 400 µl of cell lysis solution was added [1.58 g of Tris–HCl (Quality Biological, Gaithersburg, MD), 37.22 g EDTA disodium salt (Quality Biological, Gaithersburg, MD) filled to 1 L with RNase/DNase-free water, pH adjusted to 8.0 with 10 M NaOH (Sigma Aldrich, St. Louis, MO) as necessary]. We incubated samples with 60 µl of Proteinase K at 65 °C for 1 h. The resulting lysate was transferred to a clean 1.5-ml tube. We added 3.5 µl RNase A (20 mg/ml; Life Technologies; Grand Island, NY) and incubated at 37 °C for 15 min after mixing. We added 200 µl of Ammonium Acetate solution (289.1 of Ammonium Acetate per 500 ml of RNase/DNase-free water) (Quality Biological; Gaithersburg, MD) to the samples and vortexed at high speed for 20 s to mix. The samples were then incubated on ice for 5 min. The supernatant was transferred to a clean 1.5-ml tube, and we added 3 µl of glycogen (Qiagen; Valencia, CA) to aid pellet visualization. To precipitate the DNA, we added 700 µl of 100% isopropanol (VWR International; Radnor, PA). Samples were then incubated for 1 h at − 20 °C. After incubating, the samples were centrifuged at maximum speed for 5 min. The supernatant was removed, and the pellet was washed with 600 µl of 75% ethanol (NIH Supply Center; Gaithersburg, MD), then centrifuged at maximum speed for 5 min. The ethanol was removed, and the pellet was allowed to air-dry for 15 min. Samples were re-hydrated in 120 µl of RNase/DNase-free water.

We then purified the DNA with a phenol–chloroform extraction. We added 80 µl of 10 mM Tris (Quality Biological; Gaithersburg, MD), 1 mM EDTA (pH 7.8; Quality Biological; Gaithersburg, MD), and 200 µl of fresh phenol:chloroform:isamyl alcohol (25:24:1) (Sigma Aldrich; St. Louis, MO) to each DNA sample. After vortexing samples for 30 s to mix, we centrifuged them at maximum speed in a 4 °C centrifuge for 5 min. The upper layer (~ 170 µl) was transferred to a clean 1.5-ml tube. An approximately equal volume of chloroform (200 µl; NIH Supply Center; Gaithersburg, MD) was added and vortexed to mix. The sample was centrifuged at maximum speed in a 4 °C centrifuge for 5 min. The resulting upper layer (~ 150 µl) was transferred to a clean 1.5-ml tube. Next, the sample was incubated with 20 µl sodium acetate (NaOAc) (Sigma Aldrich; St. Louis, MO), 500 µl of pure ethanol, and 1 µl of glycogen. Samples were then centrifuged at maximum speed in a 4 °C centrifuge for 30 min. The supernatant was poured off each sample, and the resulting pellet washed with 500 µl of 70% ethanol. The sample was centrifuged at maximum speed for 5 min. The supernatant was removed, and we allowed the sample to air-dry for 5 min. DNA pellets were then hydrated in 25 µl of sterile buffer (10 mM Tris, 0.1 mM EDTA, pH 7.8) and heated in a 55 °C water bath for 2 min. We used the Nanodrop 8000 (Thermo Fisher Scientific; Asheville, NC) to estimate DNA concentration.

### DNA library preparation and sequencing

For each sample, one microgram of genomic DNA was sheared to ~ 350 bp using a Covaris E210 (Covaris, Woburn, MA) with settings: duty cycle 10%; intensity 5; cycles/burst 200; time 45 s. The Tru-Seq DNA PCR-Free LT Sample Prep Kit (Illumina, San Diego, CA) was used to prepare libraries according to the manufacturer's protocol. A HiSeq2500 (Illumina, San Diego, CA) with version 3 sequencing reagents generated a minimum of 47 million paired-end 126 base reads per library. Sequences were processed using RTA version 1.18.64 and CASAVA version 1.8.2.

### Sequence alignment and SNP calls

We aligned the sequences to the *D*. *melanogaster* assembly BDGP Release 6, UCSC version dm6 using with Burrows-Wheeler Alignment-MEM version 0.7.12^73^ and Novoalign version 3.02.07 (Novocraft Technologies, Selangor, Malaysia), with the -t 400 option to optimize alignment speed. Informative SNPs and indels in Freeze 2.0 of the DGRP^52^ were used as a starting point after converting the coordinates to the Release 6 assembly^74^. Reads were realigned around indels from this set of polymorphisms with GATK version 2.8.1^75^. Duplicate reads were removed with samtools version 0.1.18 after alignment, merging, and sorting of the reads. Novel variants were discovered using LoFreq version 2.1.2^51^ with default parameters. Allele counts for all single nucleotide variant sites (both known DGRP Freeze 2.0 and novel LoFreq polymorphisms) were tallied with the `bamcounts' command of the bardCNV package (http://github.com/nhansen/BardCNV) along with the -minqual 20 option to retain reads with a minimum phred quality of 20. Counts for reads spanning indels were performed by first widening indel variants to their narrowest unambiguous region, then tallying spanning reads both with and without the indel using the perl module Bio::SamTools (http://search.cpan.org/~lds/Bio-SamTools/lib/Bio/DB/Sam.pm). Confidence interval boundaries with the highest posterior density for the estimated read allele proportions were calculated in R using CRAN `binom' package's `binom.bayes' function. For downstream analyses, we considered all bialleleic polymorphisms with total coverage of at least 10 and less than 226 (the 99.5% point of the distribution)^38^. To delineate de novo mutations from potential sequence error, we required a LoFreq MaxQual score of 426 or greater, which corresponded to the 95.0% level of the distribution of known DGRP variants.

### The variance and probability distribution of random genetic drift

Under a Wright–Fisher model with constant population size *2N*_*e*_ and initial frequency *p*_0_ (and equivalently *k*_0_ copies) of an allele *A* at generation 0, genetic drift occurs by random sampling of the copies that make up the population at that time. This is the definition of sampling from a binomial distribution $k_{1} \sim Binomial\left( {2N_{e} , p_{0} } \right)$ and can be straightforwardly simulated by drawing a random value from that distribution—*k*_1_ will be the new number of copies of allele *A* in the population at generation 1, and will be an integer value between zero and *2N*_*e*_, which divided by the constant *2N*_*e*_ population size equivalently defines allele frequency *p*_1_ at generation 1. Repeating the procedure for a number of generations defines the drift trajectory of allele *A* for a single population. This procedure was replicated 10,000 times with initial frequencies *p*_0_ ranging from 0.01 to 0.5 in 0.01 increments to obtain a distribution of trajectories for each replicate of the long sleeper and short sleeper populations. The process was repeated to simulate drift on the *X* chromosome, using *N*_e_ instead of 2*N*_e_. We used 2*Ne* = 84 as the estimate of the variance effective population size, as was computed for these populations previously^38^.

The variance among trajectories as a function of the previous generation can be written in closed form^62^ and it can be recursively computed for any number of generations. The entire probability distribution obtained by simulating replicate populations as above can in fact also be computed analytically—that requires accounting for the uncertainty in the number of copies at each generation, i.e. instead of a single known value, allele frequency follows a probability distribution. This specification is that of a compound or mixture distribution, where a parameter *y* in a probability distribution is itself distributed according to an arbitrary probability distribution, and the resulting distribution has the form:

$$\begin{aligned} P\left( x \right) & = \smallint P\left( {x{|}y} \right) \cdot Q\left( y \right)dy \quad \left( {{\text{for}}\;{\text{continuous}}\;{\text{variables}}} \right)\;{\text{or}}\, \\ P\left( x \right) & = \mathop \sum \limits_{y} P\left( {x{|}y} \right) \cdot Q\left( y \right) \;\quad \;\left( {{\text{for}}\;{\text{discrete}}\;{\text{variables}}} \right). \\ \end{aligned}$$

For the sampling distributions that define the Wright–Fisher model both distributions are of the same kind: the binomial sampling probability in the current generation comes from the number of copies obtained from the previous generation, and therefore being binomially distributed as well. For the number of copies of allele *A* in generations 0, 1, 2, 3 and any number of generations, denoted *G*, and denoting the probability distribution of the number of alleles *k* at generation *i* we have:

The number of alleles in generation 0 (known): $k_{0}$

$${\text{where}}\;{\text{allele}}\;{\text{frequency:}}\;p_{0} = k_{0} {/}2N_{e}$$

then the distribution of the number of alleles in generation 1 is:

$$\begin{aligned} & B\left( {k_{1} } \right) = \left( {\begin{array}{*{20}c} {2N_{e} } \\ {k_{1} } \\ \end{array} } \right)p_{0}^{{k_{1} }} \left( {1 - p_{0} } \right)^{{2N_{e} - k_{1} }} \\ & \quad \quad \quad \quad {\text{where}}\;p_{1} = k_{1} /2N_{e} \\ \end{aligned}$$

for generation 2, the distribution is:

$$\begin{aligned} & B\left( {k_{2} } \right) = \mathop \sum \limits_{{k_{1} = 0}}^{{2N_{e} }} \left( {\begin{array}{*{20}c} {2N_{e} } \\ {k_{2} } \\ \end{array} } \right)\left( {\frac{{k_{1} }}{{2N_{e} }}} \right)^{{k_{2} }} \left( {\frac{{2N_{e} - k_{1} }}{{2N_{e} }}} \right)^{{2N_{e} - k_{2} }} \cdot \left( {\begin{array}{*{20}c} {2N_{e} } \\ {k_{1} } \\ \end{array} } \right)p_{0}^{{k_{1} }} \left( {1 - p_{0} } \right)^{{2N_{e} - k_{1} }} \\ & \quad \quad \quad \quad = \left( {\begin{array}{*{20}c} {2N_{e} } \\ {k_{2} } \\ \end{array} } \right)\mathop \sum \limits_{{k_{1} = 0}}^{{2N_{e} }} \left( {p_{1} } \right)^{{k_{2} }} \left( {1 - p_{1} } \right)^{{2N_{e} - k_{2} }} \cdot B\left( {k_{1} } \right) \\ & \quad \quad \quad \quad \quad {\text{where}}\;p_{2} = k_{2} {/}2N_{e} . \\ \end{aligned}$$

for generation 3, the distribution is:

$$\begin{aligned} & B\left( {k_{3} } \right) = \mathop \sum \limits_{{k_{2} = 0}}^{{2N_{e} }} \left( {\begin{array}{*{20}c} {2N_{e} } \\ {k_{3} } \\ \end{array} } \right)\left( {\frac{{k_{2} }}{{2N_{e} }}} \right)^{{k_{3} }} \left( {\frac{{2N_{e} - k_{2} }}{{2N_{e} }}} \right)^{{2N_{e} - k_{3} }} \cdot \mathop \sum \limits_{{k_{1} = 0}}^{{2N_{e} }} \left( {\begin{array}{*{20}c} {2N_{e} } \\ {k_{2} } \\ \end{array} } \right)\left( {\frac{{k_{1} }}{{2N_{e} }}} \right)^{{k_{2} }} \left( {\frac{{2N_{e} - k_{1} }}{{2N_{e} }}} \right)^{{2N_{e} - k_{2} }} \cdot \left( {\begin{array}{*{20}c} {2N_{e} } \\ {k_{1} } \\ \end{array} } \right)p_{0} ^{{k_{1} }} \left( {1 - p_{0} } \right)^{{2N_{e} - k_{1} }} \\ & \quad \quad \quad = \left( {\begin{array}{*{20}c} {2N_{e} } \\ {k_{3} } \\ \end{array} } \right)\mathop \sum \limits_{{k_{2} = 0}}^{{2N_{e} }} \left( {p_{2} } \right)^{{k_{3} }} \left( {1 - p_{2} } \right)^{{2N_{e} - k_{3} }} \cdot B\left( {k_{2} } \right) \\ & \quad \quad \quad \quad {\text{where}}\;p_{3} = k_{3} {\text{/}}2N_{e} . \\ \end{aligned}$$

Generalizing to generation G, the distribution is:

$$\begin{aligned} & B\left( {k_{G} } \right) = \left( {\begin{array}{*{20}c} {2N_{e} } \\ {k_{G} } \\ \end{array} } \right)\mathop \prod \limits_{g = 2}^{G} \mathop \sum \limits_{{k_{{\left[ {g - 1} \right]}} = 0}}^{{2N_{e} }} \left( {\begin{array}{*{20}c} {2N_{e} } \\ {k_{{\left[ {g - 1} \right]}} } \\ \end{array} } \right)p_{{\left[ {g - 1} \right]}}^{{k_{g} }} \left( {1 - p_{{\left[ {g - 1} \right]}} } \right)^{{2N_{e} - k_{g} }} \cdot \left( {\begin{array}{*{20}c} {2N_{e} } \\ {k_{1} } \\ \end{array} } \right)p_{0}^{{k_{1} }} \left( {1 - p_{0} } \right)^{{2N_{e} - k_{1} }} \\ & \quad \quad \quad = \left( {\begin{array}{*{20}c} {2N_{e} } \\ {k_{G} } \\ \end{array} } \right)\mathop \prod \limits_{g = 2}^{G} \mathop \sum \limits_{{k_{{\left[ {g - 1} \right]}} = 0}}^{{2N_{e} }} \left( {\begin{array}{*{20}c} {2N_{e} } \\ {k_{{\left[ {g - 1} \right]}} } \\ \end{array} } \right)p_{{\left[ {g - 1} \right]}}^{{k_{g} }} \left( {1 - p_{{\left[ {g - 1} \right]}} } \right)^{{2N_{e} - k_{g} }} \cdot B\left( {k_{1} } \right) \\ & \quad \quad \quad {\text{where}}\;p_{g} = k_{g} /2N_{e} . \\ \end{aligned}$$

The analytical calculations of the compound distribution and frequency histograms obtained from the replicate simulation trajectories are equivalent and for 2*N*_*e*_ = 84 the two ways of computing match very closely, as expected when there are enough simulations.

### Likelihood of the observed shifts under neutral evolution (random genetic drift)

With the full probability distribution of allele frequencies after a number of generations for a given initial frequency, it is possible to compute the probability of observing each shift under the neutral model. The probabilities of the allele frequency changes that we observed can then be multiplied (or rather the log probabilities can be added) to obtain a total likelihood for a set of polymorphisms in the same selection scheme, replicate, chromosome or combination thereof. The likelihood was calculated as

$$L_{12} \left( {p_{75} } \right) = \left( {\begin{array}{*{20}c} {2N_{e} } \\ {k_{G} } \\ \end{array} } \right)\mathop \prod \limits_{g = 12}^{G = 75} \mathop \sum \limits_{{k_{{\left[ {g - 1} \right]}} = 0}}^{{2N_{e} }} \left( {\begin{array}{*{20}c} {2N_{e} } \\ {k_{{\left[ {g - 1} \right]}} } \\ \end{array} } \right)p_{{\left[ {g - 1} \right]}}^{{k_{g} }} \left( {1 - p_{{\left[ {g - 1} \right]}} } \right)^{{2N_{e} - k_{g} }}$$

where $L_{12} \left( {p_{75} } \right)$ is the likelihood of observing an allele frequency $p_{75}$ after 62 generations of relaxed selection (i.e., from generation 12 through generation 75). This expression is obtained directly from the expression for $B\left( {k_{G} } \right)$ by noting that indices *g* are arbitrary labels and the first generation can be labeled g = 12, and can be included into the product by acknowledging that the sum is over a distribution where 100% probability mass is on $p_{12}$ in the generation. Therefore, under this model of neutral evolution, a group with higher likelihood than another is more compatible with genetic drift alone producing the observed pattern. Moreover, the ratio between probabilities quantifies how much more likely that is.

### Average likelihoods of significant set of variants and distribution of neutral sites

The sum or average likelihood was computed for each replicate population, and compared; alternatively, they can be compared to sets of sites not found to be significant (“random sets”). To construct these sets, sites were randomly chosen in the same number (126) and chromosomes where the variants of interest occurred. One thousand random sets were chosen per replicate population, and the average likelihoods computed for each of them in the same way as for the variant of interest, resulting in a distribution of these values for putative neutral sites. If the average likelihood of a replicate population falls well within the distribution of the random sets, it is unlikely to be different from the average variant.

Using the compound distributions described above likelihoods can be computed for each site found to be significant (“variants of interest”). The frequency of variants that are fixed or lost cannot change, and therefore have probability one of staying the same; however, a variant at very high or low frequency can appear to be fixed or lost, respectively, due to sequencing noise. In this case there is a nonzero probability that the frequency changes; therefore if a shift is observed the variant must not have been fixed or lost, and we assume the initial frequency was the lowest possible (0.01) or highest possible (0.99), respectively, and compute the likelihood of the shift based on that initial frequency.

### Identification of de novo mutations

We examined the sequence data for potential de novo mutations. First, we checked to see whether the variant had been previously identified in DGRP^52^. Of the non-DGRP variants remaining, we eliminated potential de novo mutations of low quality by: 1) eliminating variants with LoFreq quality scores lower than the 95% level (124), and 2) eliminating variants with very low or very high total numbers of reads (reads below 40 (0.5% level) or above 226 (95% level). We required potential de novo mutations to have reads that were unique to both samples for a given replicate population (i.e., DNA samples 1 and 2 of the Long sleep Replicate 1 population have the variant, while no other populations have the variant). We also ensured that de novo mutations did not arise in the prior artificial selection study at any generation^38^.

## Supplementary information

Supplementary Information 1.

Supplementary Information 2.

## Data Availability

Raw DNA sequence data from generation 75 of the selected populations/unselected controls and Sleep Advanced Intercross Population sequence data from generation 102 have been deposited in the NCBI SRA database under BioProject PRJNA588984. Raw DNA sequence data from generation 12 have been deposited in the NCBI SRA database under BioProject PRJNA369048. All remaining data are within the manuscript and its supporting information files.
