# Supplementary material for: Natural selection on sleep duration in Drosophila melanogaster
Source: Sci Rep. 2020 Nov 26;10:20652. doi: 10.1038/s41598-020-77680-0 (PMC7691507; doi:10.1038/s41598-020-77680-0)
Supplement: Supplementary file 1 — Supplementary Information 1. [file 41598_2020_77680_MOESM1_ESM.pdf]

Natural selection on sleep duration in *Drosophila melanogaster*

Caetano Souto-Maior, Yazmin L. Serrano Negron, Susan T. Harbison

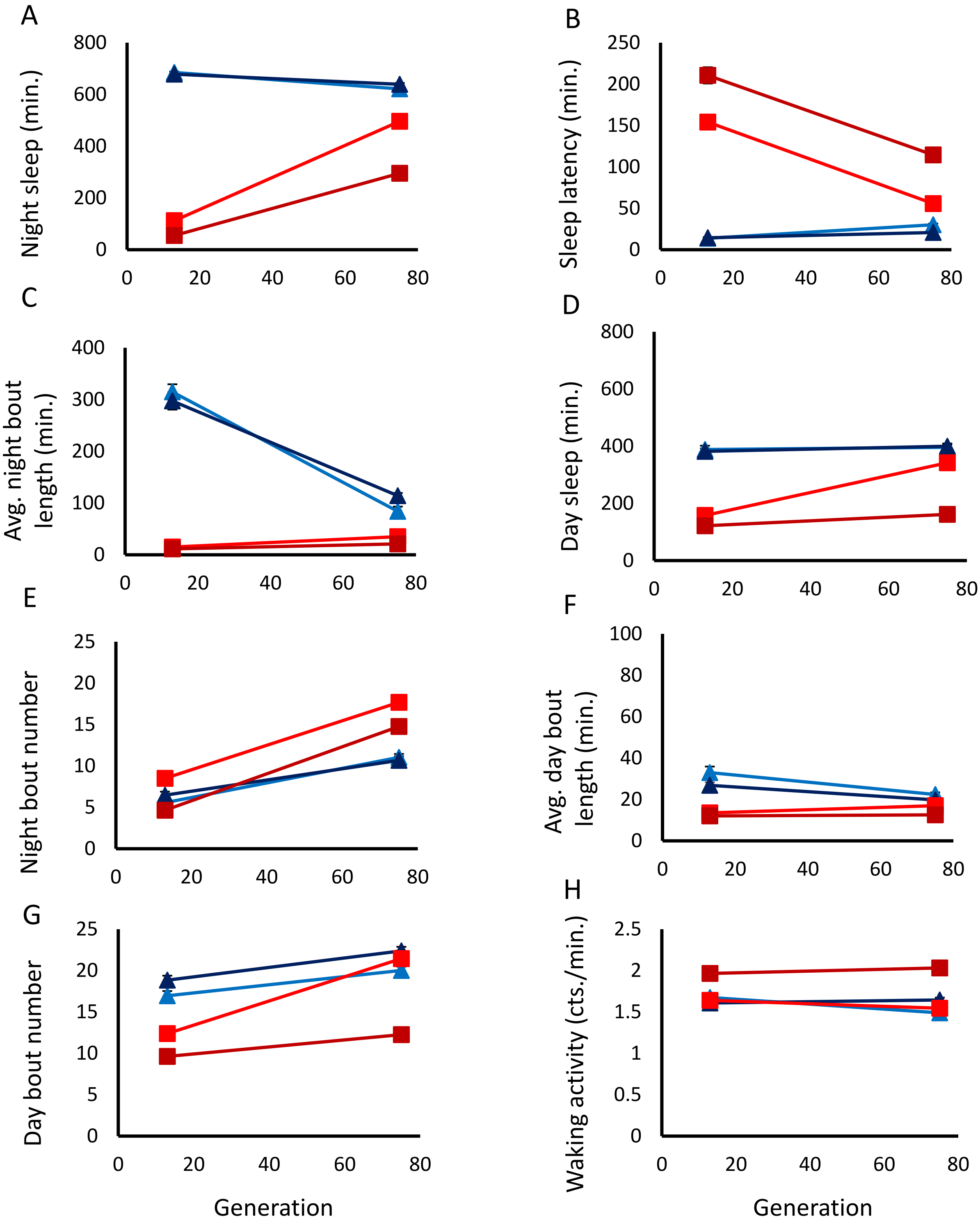

Supplementary Figure S1. Correlated responses to relaxed selection. The graphs show the change in sleep traits observed from Generation 13 to Generation 75. *P*-values represent a significant selection × generation interaction; see Supplementary Table S1 for the full model parameters. (A) Night sleep (*P* = 0.0357); (B) sleep latency (*P* = 0.0021); (C) avg. night bout length (*P* = 0.0136); (D) avg. day bout length (*P* = 0.0318); (E) night bout number (*P* = 0.0268); (F) day sleep (*P* = ns); (G) day bout number (*P* = ns); and (H) waking activity (*P* = ns). Light and dark red squares represent short sleeper replicate populations 1 and 2 respectively; Light and dark blue triangles represent long sleeper replicate populations 1 and 2 respectively.

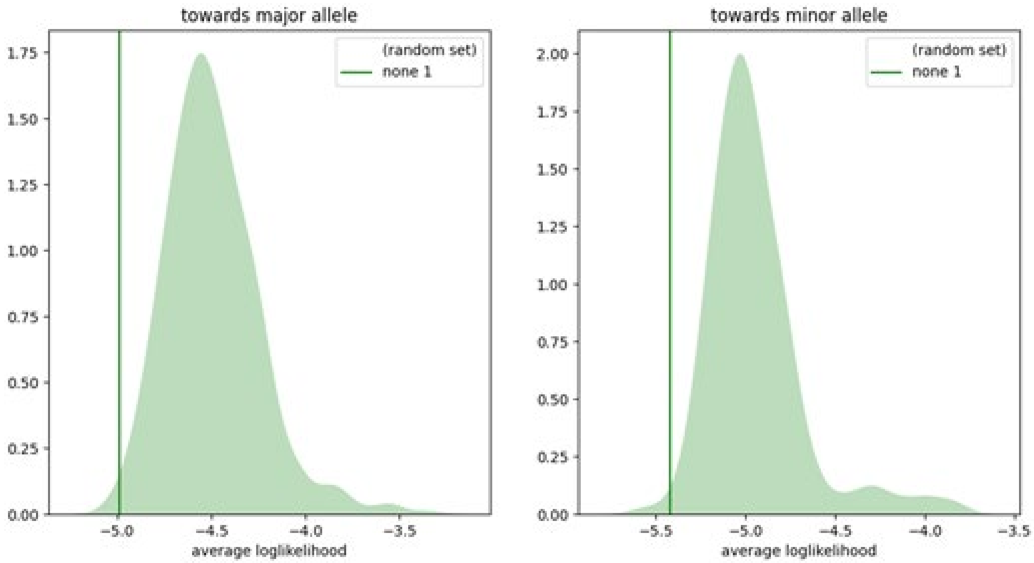

Supplementary Figure S2. Sleep Advanced Intercross Panel (SAIP) average loglikelihood. The average log likelihood for the SAIP for random drift is plotted relative to the distribution of loglikelihoods of random drift obtained by sampling 126 random polymorphisms 1,000 times from Generation 0 and Generation 102. Allele frequencies are split according to those that changes towards the major allele in the intervening generations, and those that changed toward the minor allele.
